# Signs in People with Intellectual Disabilities: Interviews with Managers and Staff on the Identification Process of Dementia

**DOI:** 10.3390/healthcare6030103

**Published:** 2018-08-25

**Authors:** Göran Holst, Maria Johansson, Gerd Ahlström

**Affiliations:** 1 The Swedish Red Cross University College, Box 1059, SE-141 21 Stockholm, Sweden; Goran.Holst@rkh.se; 2 Department of Health Sciences, Faculty of Medicine, Lund University, Box 157, SE-221 00 Lund, Sweden; maria.johansson@med.lu.se

**Keywords:** intellectual disability, mental retardation, learning disability, older people, dementia, signs of dementia, frailty, qualitative study, interview study, caregivers’ experiences

## Abstract

The life expectancy of people with intellectual disabilities (ID) has steadily increased, which has been accompanied by an increased risk of dementia. Staff and managers are key resources for safety diagnosis since they deliver information about people with ID behavior every day. The aim of the present study was to explore the identification process employed by staff and managers to detect signs of suspected dementia in people with an ID within intellectual disability services (ID-services). Twenty managers and 24 staff within an ID-service were interviewed and qualitative latent content analysis was applied. A model consisting of three themes on three levels of resources for the identification process of signs of suspected dementia emerged from the analysis. On the first level was the time and continuity in the care relationship, which is crucial for identifying and responding to changes in cognitive ability that indicate dementia. On the second level, the staff identify deficiencies in their own knowledge, seek support from colleagues and managers within their workplace and, on the third level, outside their workplace. Staff and managers expressed a need for early and continuous guidance and education from specialists in dementia and primary healthcare. This finding indicates an urgent need for intervention research and digital support for staff in dementia care.

## 1. Introduction

Aging people in the general population and dementia prevalence are increasing [1], as well as for people with an intellectual disability (ID). [1]. ID is typically a life-long condition with onsets occurring before the age of 18 years characterized by significant limitations in intellectual and cognitive functioning as expressed in reasoning, learning, problem solving, and behavior adaptive skills [2,3]. Aging people with an ID have a high incidence of dementia and, especially among people with Down’s syndrome, dementia presents early [4,5]. When people with an ID are aging, many of them are cared for in community residential care [6]. The characteristics of developing dementia in a general population are mainly stated in the form of increasing memory difficulties, language difficulties, and, hence, difficulties in coping with many of the challenges of everyday life. Recognizing these changes in people with an ID is even more complex and implies several challenges [7,8] in the sense that they already have a cognitive impairment and linguistic difficulties. Even depression and anxiety, which are common in the early stages of dementia, are more difficult to detect in people with an ID [8]. Previous research about dementia among older people with ID comprised issues regarding epidemiology, assessments tools for diagnosis, and management in the form of pharmacological and non-pharmacological treatment [9]. However, assessment for early identification of dementia can be complicated due to a lack of well-established instruments adapted to people with ID, which also had comprehensive information about reliability and validity. The most instruments used to diagnose dementia are based on people who previously had intact cognitive functioning. Due to the pre-existing decreased cognitive abilities in the population with ID, the early detection of dementia is challenging. However, accurate screening for diagnosis of dementia is crucial in order to provide appropriate interventions, care, and support as early as possible [10,11,12,13,14].

People with an ID have the same right as others for an investigation based on the individual’s needs and ability when they show signs of dementia [15]. However, a barrier to meeting this requirement in practice is the absence of a clear structure and methodological support for the implementation of a dementia investigation adapted to people with an ID. According to The British Psychological Society [16], there has been an awareness for several years about dementia mainly in the general population and a proliferation of strategies and standards documents have ensued. However, dementia among people with an ID has still received minimal focus. One possible reason for this is the diagnostic challenges due to the complexity involved in detecting the early signs of dementia in a person with an ID, which is mentioned above. However, Moran and colleagues [17] focus on an assessment in several steps conducted using national recommendations for the evaluation and management of dementia. This includes a medical examination, review of medications, baseline functioning, current functioning, and documentation of family and social history. Therefore, a family member or caregiver have, along with the medical specialist, a decisive role in such an investigation due to their knowledge about the history of the person with an ID [17].

Staff and managers are key resources for supporting dementia care in the early stages of screening and diagnosing dementia by delivering information about the person with an ID who lives and is cared for in residential care [8]. It is well known that people with an ID may have difficulty seeking care, according to their actual needs. One of the reasons may be the difficulty to express their wishes and needs in a manner that is understandable to others. Furthermore, because of their ID, they may show signs that may be similar to cognitive difficulties that develop in dementia patients, which may complicate the investigation and further planning for adapted care [8]. Based on findings from an explorative study conducted by Cleary and Doody [18], knowing the person well is a key element in providing a mode of care that supports and comforts the person with an ID or dementia, i.e., providing a mode of care according to actual needs. In a review of the literature by Cleary and Doody [19], it was found that a professional caregiver considered caring for people with an intellectual disability and dementia sometimes challenging, stressful, and time consuming and believed that professional caregivers required further knowledge and educational training concerning all stages of dementia. It was also found that the ability of staff to seek advice and support from multidisciplinary professionals and management appears to be determined based on successful coping and an ability to handle these challenges [19]. 

In addition, leaders greatly influence the quality of the services and care delivered to people with an ID. Studies have shown the importance of leaders in organizations and their indirect influence on outcomes [20] through their leadership of the staff [21]. The development and the support of staff need to focus on the service users’ quality of life, how and when support is delivered, and the organization of meaningful activities for the service users [21]. Taking into consideration the important role of staff and managers for the person with an ID in the early stages of dementia, their experience should, therefore, be explored and can thus provide a source of knowledge about how signs of dementia can manifest in everyday life. This knowledge may lead to continued development of a person-centered care aimed at persons with an ID and dementia. Therefore, the aim of the present study was to explore the identification process employed by staff and managers to detect signs of suspected dementia in people with an ID within intellectual disability services.

## 2. Materials and Methods

The design of the study was an explorative qualitative semi-structured interview study with staff and managers within the intellectual disability service for people with an ID.

### 2.1. Study Setting

The Swedish welfare system is mainly public, largely funded by taxes, and designed to provide everyone with equal access to health care, elderly care, and disability and social services based on each person’s need for services, support, and care. The responsibility is shared between 21 county councils (primary health care and specialist health care) and 290 municipalities (elderly care, disability, and social services). Since 1994, there is an entitlement law in Sweden named the *LSS* (Act Concerning Support and Service for People with Certain Functional Impairments), which is based on the Human Rights Convention [22,23] and for people with extensive and permanent functional impairment. This Act guarantees that people with comprehensive disabilities have the right to the same living conditions as the general population, which ensures support in daily living and the capacity to influence the received support. A person who fulfill the criteria of the LSS Act can apply for support, personally or with support from someone else, and any decision on a measure under the LSS can be appealed at the County Administrative Court [15]. The eight support services for adult persons with an ID, according to LSS, are: Residence with special services for adults or other specially adapted housing, daily activities, counseling and other personal support, personal assistance, companion service, contact person, relief service in the home, and a short stay away from home [22].

The current Swedish policy for people with a disability as well as for older people in the general population is “aging in place”, which means enabling people to continue to live in their own homes for as long as possible and providing assistance in accordance with their needs [24]. A group home is a private-residence located in a block of flats or a house in ordinary housing areas with special services or specially adapted accommodation for people with an ID [15]. Residents have their own small flat and share common areas such as a dining room and a large kitchen. Each group home houses 6 to 8 people from 18 years of age or older with different levels of ID. They receive the support needed in daily living from the staff at the group home who are available 24 hours a day. The residents pay a plausible fee for accommodation, recreation, and cultural activities but must be left with a sufficient amount of money for personal use.

People with ID have a right to participate in daily activity centers up to five days a week from the age of 18 to the age of 67. Activities at the daily activity centers give people with ID who are not working in the labor market nor studying some interesting and meaningful, day-to-day structured activities. The daily activity centers provide various types of activities from more open social cohesion to more structured employment adapted to the needs of each individual. It is mandatory for the municipalities to provide intellectual disability services (ID-service) in Sweden in accordance with the *LSS act* [15,22].

### 2.2. Sampling and Participants

#### 2.2.1. The Selection of Managers

The study included staff and managers working in an ID-service in a Southern city in Sweden. The inclusion criterion for managers to participate in the study was that they have had experience leading a group home or daily activity center where older residents 55 years of age and above live or have lived. The inclusion criteria for staff attending the study were at least one year of experience in working in one of these organizations and experience in providing support to older persons with IDs aged 55 or above. The first-line managers in the municipality were in general responsible for two to three group homes or daily activity centers while the second-line managers were responsible for all first-line managers within their designated area of the town.

All 12 second-line managers in the current city were contacted by e-mail with an informative letter about the study and a request to participate in the study if they fulfilled the inclusion criteria. The second-line managers then contacted the first-line managers within the relevant responsibility area with information letters and a request to participate in the study. There were four second-line managers and 16 first-line managers who judged that they met the criteria and accepted to participate in the study. These managers were included in this study. Fifteen of the 20 managers worked in group homes and five worked in daily activity centers. Five were men and 15 were women. To avoid the risk of revealing the four second-line managers’ identities, no more background data were collected about the participating managers.

#### 2.2.2. The Selection of Staff

The 16 first-line managers were given an information letter to hand to the staff in the group homes or the daily activity centers where they work as managers and asked whether they were interested in participating in the study. Twenty-seven staff wanted to participate in the study. Two of them were dropouts because of long sick-leave and one for unknown reasons. Thus, 24 staff were included in this study. Seventeen of the 24 staff worked in group homes and seven worked at daily activity centers. Four were men and 20 were women. The age of the staff ranged from 24 to 65 years of age with a mean age of 48 years (SD 10.4). They had between 2 and 41 years of experience working in a group home or a daily activity center and 75% of them had more than nine years of experience. Four of them had a university level education, six had a post-secondary education (post-gymnasium), 13 had two or three years of secondary education (gymnasium), and one person had elementary education.

Thus, the total study group was comprised of 44 participants.

### 2.3. Data Collection

The interviewer (MJ) contacted the managers and staff and asked about the willingness to participate after given information about the study and a time for the interview was decided. Just before the interview, the information about the study was repeated orally and the participants were given the opportunity to ask questions before signing a written informed consent. All interviews took place at the workplace of the managers and staff outside of the place in which people with IDs have their activities. 

A semi-structured interview guide was used in the 44 interviews, which had two main questions: What does aging mean for a person with an ID? and How do you perceive the need for support, service, and care in aging people with an ID? Follow-up questions were asked depending on how comprehensively the interviewee had answered the main open questions. The interviews were taped digitally and transcribed verbatim including pauses and emotional expressions. In the interviews emerged rich narratives that revealed that aging with ID for managers and staff meant having concern about providing service to persons who they believed had dementia. Thus, this became the focus of this study.

### 2.4. Analysis

The analysis was a qualitative latent content analysis [25,26], which was followed by an inductive approach in which codes abstracted into themes were made. The analysis was provided in the structure of Preparation, Organizing, and Reporting [25]. The Preparation phase started with reading the transcribed interviews several times to obtain a sense of the wholeness of the content. Then the units of meaning about changes in the everyday life of the person with an ID were selected from the interviews, which included signs of dementia and descriptions of the strategies of staff and managers in understanding and acting to provide care based on actual needs. In the next step, Organizing, these units of meaning were condensed, i.e., the text units that describe, in different ways, the possible signs of dementia and/or strategies for care and support. The condensed units of meaning from staff and managers were then labeled with codes, which describe the content of the text. The units of meaning, the condensed units of meaning, and the codes with similar content were read several times and, from patterns of core content, the themes were developed. The Organizing phase ended with thorough discussions by all the authors together in several meetings about the result of the analysis and the themes were refined. Reporting means to describe the results of the analysis process by illustrating a conceptual map [25] with three themes and with illustrated quotations.

### 2.5. Ethics Approval and Consent to Participate

Before starting the study, the responsible health care and social welfare manager in the current city as well as the Regional Ethics Review Board in Lund, Sweden (reference number: 2013/83) approved the study. The research was guided by the research ethical principles in the Declaration of Helsinki and the participants gave their oral and written informed consent before the interviews. To respect the participants’ autonomy, all the participants were informed that they had the right to withdraw from the study at any time without any consequences. To maintain the principle of non-maleficence, the participants were guaranteed confidentiality, which was taken into account in data collection by coding the interviews and presenting the data on the group level.

## 3. Results

**Figure 1:**
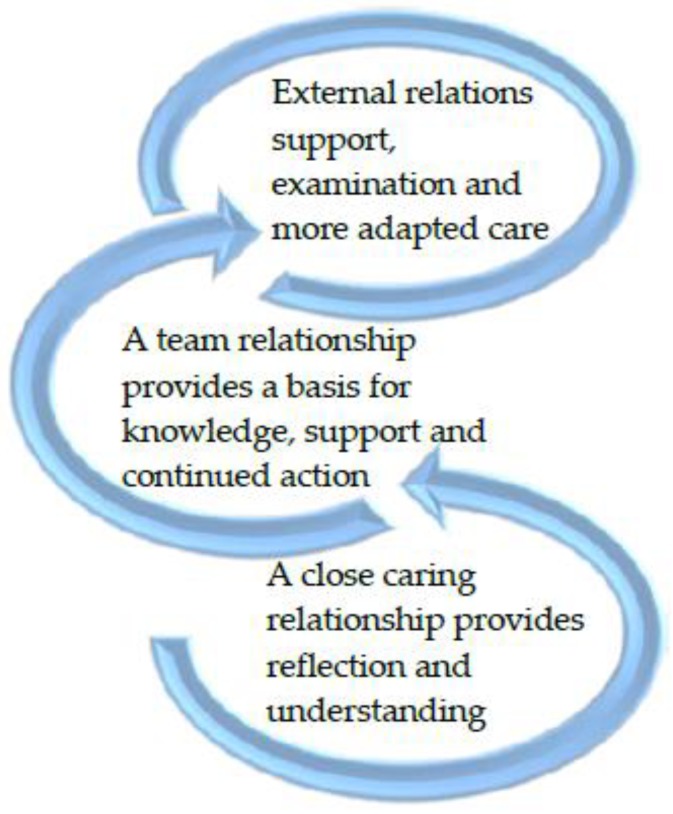
A model of the three levels of resources for the identification process of signs of people with an ID and suspected dementia.

Three themes emerged from the data and these constitute three levels of the identification process of signs when dementia is suspected in a person with an ID. The three themes are known as: A close caring relationship provides reflection and understanding, a team relationship provides a basis for knowledge, support and continued action, and external relations support examination and more adapted care (Figure 1). Each theme was generated from patterns of core content (see Table 1).

### 3.1. A Close Caring Relationship Provides Reflection and Understanding

The described actors in this part of the caring process consist of the person with an ID and staff (Figure 1, Table 1). Under this theme, the staff clearly describe how time and continuity in the care relationship are crucial to being able to identify and respond to the often-subtle changes in behaviors, which can indicate signs of a change in cognitive ability and thus indicate dementia.

*“I can’t say how many years ago it was. His behavior just changed completely and we didn’t understand a thing. He’d been the nicest man in the world and then he just changed completely.”*

(Member of staff, group home, female)

A care relationship based on good conditions provides guidance on how treatment and support need to be adapted, according to changes in needs and new circumstances, since time and continuity allow staff to detect changes. The participants described a long-term caring relationship and how changes that mark possible dementia usually requires staff who strive to understand the person’s changed expression and observed behaviors.

*“A person’s memory gets worse. But if you know what it’s been like over time, you can see that there’s a new situation.”*

(Member of staff, daily activity center, female)

The transition from a caring relationship, without signs of dementia, to a relationship where such signs are detected will affect both parties. Everyday life and its activities are affected. It can be forgetfulness that makes it harder for the person with an ID to orientate in time and finish a meal. For the staff who should provide the support, the change requires an understanding of what is happening and, based on this, the insight that efforts need to be adapted and how to adapt. The staff expressed a compassion and concerns over changes in the behavior and everyday habits of the person. Based on this sympathy, they needed an internal reflection by seeking to understand what the changes lead to and how treatment and care can best be adapted. They express how it is a challenge to see signs of dementia since it manifests in behaviors like irritation, aggressiveness, forgetfulness, and confusion rather than being spoken in linguistic terms.

*“There are two whose behavior indicates dementia, though not quite in the same way. The fact that they don’t talk makes things a bit more difficult, of course. But you do notice changes in behavior especially in the case of one of them. For instance, you’ll suddenly find him standing in the shower at four in the afternoon, which hasn’t happened before. He has a shower in the morning and he’s always been careful to keep to that routine.”*

(Member of staff, group home, female)

This imposes other demands on the staff’s responsiveness compared to when someone expresses their forgetfulness in words. The staff emphasize how continuity in contact is an important part of being able to “feel needs that are expressed as behaviors” among persons with IDs. It also appears that staff are uncertain about how a changed behavior of the person with an ID should be interpreted as signs of dementia, signs of normal aging for people with IDs, or signs of symptoms of other bodily disorders such as an infection or pain. Necessary knowledge about normal and abnormal aging is, therefore, needed.

*“But you have the same problem with people with dementia who don’t talk. A person can hit themselves and it can be a sign that they’re in pain. And I think, I really do think, that we all need to know what happens when we get older.”*

(Member of staff, group home, female)

### 3.2. A Team Relationship Provides a Basis for Knowledge, Support, and Continued Action

The described actors in this second part of the three-step level consist of staff and managers in their own workplace (Figure 1, Table 1). According to staff, it is necessary in many cases to obtain support from colleagues, which can lead to a proper investigation of the changes they discover in the person with an ID. This indicates either an onset of dementia or the expression of a challenging behavior, which is difficult to master. The staff explains how to transfer their experiences and reflections from the long-standing and ongoing process together with the person with an ID to colleagues and managers in recurring meetings. By doing so, they can gain understanding and relief as well as confirmation of their interpretation of a strange act of suspected dementia or insights that can lead to an understanding of how to respond differently.

*“You’ve got to take the situation as it comes, so to speak. And then, of course, we talk a lot about it in the staff group, so that everybody’s alert to the fact that something’s happened with regard to the person. As when one person went around picking up this, that and the other—collecting little things. She hadn’t done that before.”*

(Member of staff, daily activity centre, female)

The staff emphasizes the importance of having this forum and how it enables a changed action in care, which may be the best available to the person with an ID. The staff expresses how managers have a responsibility and an opportunity to provide support based on their knowledge and experience. The managers, in turn, received descriptions from the staff of situations in which changes in behavior have occurred and they try to find possible internal support methods.

*“Well, what made the staff start to suspect dementia was that the person became confused and couldn’t find the way home and …”*

(Manager, group home, male)

The person with an ID may, because of a cognitive impairment and possible dementia, develop a radically changed need for support in his everyday life. The changed behavior affects the individual himself but also his surroundings in the form of other residents and staff. An experienced problem was when other persons living at the group home could not handle the changed behavior associated with dementia. For managers, it is common to think about a change of housing and/or the adaptation of the workplace. They express a willingness to let, as far as possible, the person with an ID stay in their current residence and compensate with more customized support to make this possible. There were different opinions among managers and staff and between managers as to whether it was better for the older person with dementia to stay at the group home or to move to an ordinary group home for older persons with dementia. If the person had lived at the group home for a long time and felt secure and comfortable there, the main opinion was that it was better for him/her to stay and adapt to the environment.

*“I mean, it’s where they live, and if they feel a sense of security there, you mustn’t make them move just because they’re older and you can’t make somebody move because they suffer from dementia.”*

(Member of staff, daily activity center, female)

Another opinion was that it could be better to move the person with ID to an ordinary group home for older people with dementia when the staff have not had enough experience and do not have sufficient knowledge about dementia care. A leader gave an example of a group home for older people with dementia, but it did not turn out well for organizational reasons. From the interviewees, it appears that there is no optimal housing that suits those with ID and dementia.

*“Is it dementia that should determine where he’s going to live or is it quality of life? It’s maybe more important that he’s here where he knows the staff and his neighbors and the environment and the area. Yes, that’s maybe more important.”*

(Member of staff, group home, female)

There is, however, a desire of managers for staff to have better education regarding dementia in the ordinary group home instead of adapted housing, so that future demands of the growing group of older persons with an ID who are developing dementia can be met.

*“I don’t think the person would have been better off in an ordinary home for people with dementia. On the other hand, the staff at the group home do need greater competence concerning dementia.”*

(Manager, group home, female)

### 3.3. External Relations Support Examination and More Adapted Care

The described actors on this third level consist of the staff and managers in the ID-service, external staff from other municipal units, and primary health care and specialist care (Figure 1, Table 1). Managers pave the way for contact with external resources in the form of support from, for example, nurses specially trained in dementia care for educational efforts and skills development, but they can also assist in finding ways to employ external actors from municipal, primary health care, or specialist care. Specialists, outside their own group, can support examinations and education.

*“In connection with a person being diagnosed as having Alzheimer’s, this wonderful nurse came here one morning and talked to us all. Very committed. She’s attached to the primary healthcare center where the person with Alzheimer’s is registered. She’s fantastic and so is the doctor.”*

(Member of staff, daily activity center, female)

In cases where the primary healthcare nurse who is connected to the group home found that the staff needed more knowledge about dementia and specialist support, a dementia nurse was consulted. If there occurred difficulties in the care of the person with an ID and dementia at the end of life such as anxiety and screaming, the staff could experience frustration and problems in handling the situation.

*“It felt as if nothing we did was right, but then the dementia nurse was an enormous help, simply by being able to put it into words and showing it was normal. She said there was nothing more we could do.”*

(Manager, daily activity center, male)

However, the availability of consultation with dementia nurses varied in different areas of the actual city. There could have been a dementia nurse previously available but not at the moment. The dementia nurses were employed in the community and not especially in the ID-service, which means their knowledge about people with IDs was limited. Support from external specialists was important at the end of a dementia progress especially in the phase with palliative care, which could be tough on the staff. Other than support from the staff group and manager, the staff could get support from nurses and physicians from the primary healthcare field on how to care for the older person at the end of life.

*“Our nurses from this part of town were brought in and her own general practitioner made regular calls towards the end. We worked together in the palliative care when the person was dying. This gave me a firm sense of security.”*

(Member of staff, group home, male)

The managers’ outline the importance of an environment that is well adapted to the changing abilities and needs of the person with an ID, which can, for example, include the support of an occupational therapist. The advice could be to consider using colors and lights.

*“When we furnish a flat in co-operation with the person, what do we have to think about? No dark mats on a light-coloured floor, for instance… You don’t want to feel as if you’re falling into a hole! And the curtains mustn’t be made of a material that reflects the light … You don’t want a situation where the person can’t really see whether there’s someone standing outside the window or not, or where, when it’s dark, the artificial light is reflected. These are things you don’t think about until they’re pointed out to you.”*

(Member of staff, group home, female)

For the most part, the staff as well as the managers experience a lack of support in diagnosing the condition in accordance with the guidelines and rights relevant to dementia. Managers expressed that there were difficulties in conducting an investigation into suspected dementia. This was mainly due to five factors: (1) the physicians at primary healthcare and other outside specialists did not have enough knowledge concerning dementia in persons with an ID; (2) a lack of developed methods to measure changes in people who already had dysfunctions; (3) a lack of knowledge in the ID-service of supportive intervention in dementia care is directed to a person with an ID; (4) a negative attitude from healthcare staff to an ID; and (5) an ethical and legal question of subjecting persons to examinations that they did not understand or want. One suggestion to facilitate the investigation was to do it at the person’s home instead of at the primary healthcare center. There was also a major issue of what benefit the person with an ID received from an investigation and dementia diagnosis. The changes in, or loss of, abilities would, nonetheless, be compensated and treated by more support from the staff.

*“What can you ask of them and how important is it? It differs from one person to another. The staff must always adjust to the person’s individual needs; find what aid or appliance the person requires at the particular time.”*

(Manager, group home, female)

It appeared that physicians in primary health care centers had difficulties in establishing a dementia diagnosis for people with an ID and it was important that staff who knew the older person with an ID well accompanied them to the office of the physicians. The staff could describe the changes in the behavior and deliver information about the older persons’ normal skills and capacities that he or she had a year ago.

*“Of course, the person needs to be accompanied by a member of staff that works closely with them. If the person goes unaccompanied to a healthcare center, the examination can be just who knows what because the dementia isn’t visible. All you see is the intellectual impairment. It needs to be made clear how the person functioned until a year ago before we started thinking that something was not as it should be.”*

(Manager, daily activity center, female)

## 4. Discussion

The interviews with staff and managers revealed that dementia is a main concern in aging for people with an ID, which, in this study, is expressed in a model of three themes on three levels of resources for the identification process of suspected dementia signs. The first theme illustrates a level where caregivers, through their everyday contact, can get information about early signs of possible dementia. In the next step, this knowledge is discussed with colleagues and managers and, lastly, managers can consult external resources to further investigate their suspicion of dementia and consider the need of staff for education and support. The study points to the need for skills development of staff who work close to the person with an ID and the shortcomings in methods, forms, and procedures for the implementation of an adequate dementia investigation for persons with an ID. Additionally, the study points to the difficulty in deciding on an accommodation and environment that is optimally adapted to the target group, i.e., aging in place with the continual development and adaptation of the staff’s skills and the environment or the being moved to adapted accommodation.

The time and continuity of the relationship between the person with an ID and staff as well as knowledge support from colleagues and managers were considered as important prerequisites for the detection of possible signs of dementia and the adaptation of the caring process in the interviews. Northway [27] argues that staff who are close to the person with ID have a unique knowledge of changes in health conditions and, therefore, points to the importance of respecting the knowledge that staff have access to. Llewellyn [28] indicates that the unique knowledge of staff represents a crucial prerequisite for starting a care process designed to discover dementia and adapt support based on changing needs. Additionally, Cleary and Doody [18] point to the fact that knowing the person with an ID and dementia is key to providing care based on individual needs and they point to proactive planning that supports the carers and, as an effect, also the person with an ID. This is also in line with, for example, Moran et al. [17] who state that a standard diagnostic workup in the evaluation and management of dementia for a person with an ID includes a detailed history that is presented by someone who is well-acquainted with the individual such as, for example, a family member or caregiver. Staff who participated in this study demonstrated a high level of commitment and motivation for their work, but they also expressed the desire for more knowledge and education as a prerequisite for continued support in line with the progression of dementia and increased need for care. The managers and staff working in an intellectual disability service generally have a social or pedagogic education, which means that they may have the same level of knowledge about dementia as the population in general regardless of any experiences they get through their work. A previous interview study from the UK shows that people in general expressed uncertainty about the illness trajectory, boundaries between age-related memory decline, cognitive impairment, and dementia [29].

Janicki [30] argues that constructive staff education and training are crucial components for the quality care of people with IDs and dementia. Thus, personalized training for staff and a proactive approach from managers where education is planned and adapted to the changing needs of caregivers is important. Managers have a key role [8] in organizing work so that space is provided for a continuous dialogue between staff in which they can discuss individual cases and problems with the inclusion of collegial support or supervision from an expert, i.e., a nurse specialized in dementia care. This form of support is presented as very important to better understanding, for example, a changed behavior but is also found to have an emotional relief function through the confirmation from colleagues [31]. However, staff does not always have knowledge about the changes that are relevant for the development of dementia or the effectiveness of any treatment and they, therefore, have difficulty participating in dialogue with other professionals. Furthermore, educational interventions may improve the awareness of mental health problems such as depression and anxiety. Costello and colleagues [32] and Tsiantis and colleagues [33] found a significant improvement in the knowledge and attitudes of staff working with persons with IDs and thus better prepared them to differentiate better various mental health problems after receiving the educational intervention.

The findings from the study also show that further support is needed and emphasize the manager’s responsibility to consult external resources when early signs of dementia can be a suspected diagnosis and the need for support increases. Actors outside of the workplace of the managers and staff such as general practitioners, occupational therapists, and registered nurses specialized in dementia care, are important resources as dementia progresses over time, but other means may also be considered. Research showing promising results using a digital diary method such as the Experience Sampling Method (ESM), which was described by Knippenberg et al. [34] to assess caregiver functioning, has emerged. The method is mainly designed for spousal caregivers to persons with dementia, but, since the relationship between professional caregivers and individuals with IDs and dementia is often long-term, it should be possible to develop similar digital methods that are refined and adapted to this caring relationship. This type of a digital diary method may provide an opportunity to better understand the everyday life of both caregivers and persons with dementia and it offers possibilities for adapting support based on the needs of the person.

The findings also indicates an opinion of staff as well as managers that the person with an ID should be offered a specialized assessment and investigation of possible dementia. However, sometimes the experts due to the experiences of staff and managers in the present study had a lack of knowledge about methods of dementia investigation and support targeted at a person with an ID and sometimes they also reflected a somewhat negative attitude towards the value and significance of special dementia investigation for this group of older people. It is well known that the diagnosis of dementia is complex in adults with ID due to their intellectual, psychosocial deficits and, most often, atypical presentation [7]. However, in recent years, several instruments have been developed, which are available or under development. For example, international experiences from special memory clinics for persons with IDs and possible dementia [4] in which a system using informant-based tools as well as objective-based tools for measuring possible dementia and evaluating the dementia process is proposed. The so-called Dementia Care Mapping (DCM) is widely used in general dementia care and may benefit care workers as well as people living with dementia in care settings [35]. DCM has been designed to improve the quality and effectiveness of person-centered care and it can be used either as an assessment of residents’ well-being and quality of life or as an outcome measure of intervention [35,36]. Schaap and colleagues [37] recently evaluated a modified form of DCM for use in ID-care and found it to be an appropriate and valuable method to support staff in their work with aging people. There are interesting examples to consider and it is a challenge for healthcare to implement any of these systems to make care more equal even for people with an ID. However, these instruments are not tested with regard to their validity and reliability and there is a need for psychometric studies in further research.

The findings of the study also deal with the question of the rights of people with IDs and dementia with respect to the choice between aging in place or moving to an adapted accommodation for dementia in general [15,22]. The staff and managers stress several possible benefits of staying in the environment where the person feels at home and the environment and treatment are adjusted as far as possible based on changing needs. Wilkinson and colleagues [38] argue that support for the staff in terms of strategic deployment of staff, i.e., extra staff in cases of disturbing behavior, is important and thus organizational flexibility is also important. This requires a good dialogue between staff and managers, which would allow many situations and problems to be solved within the staff team and the well-being of the person with an ID as well as the caregiver to be supported. However, besides dementia, this is also applicable in the case of multi-morbidities such as epilepsy, depression, vision impairments, and hearing impairments [8,39,40,41]. This means that, in addition to the increased needs arising from dementia, the staff must also handle problems related to multi-morbidity and the connected polypharmacy [42,43], which creates a great demand for knowledge and support concerning how to handle such problems when a decision has been made to let the person age in place. The development and evaluation of adapted housing to meet future demands as the group of persons with IDs and severe dementia increase is apparently an important area for further research.

The interviewees experience a diagnosis of dementia in people with IDs as problematic and as a question of the necessity for persons who do not understand or ask for the investigation. One of the ethical issues in clinical care, according to WHO [44], is “What criteria should be used to assess whether a patient has the capacity to make his or her own decisions about treatment.” It is an important ethical issue concerning, on the one hand, the same rights of health and healthcare as the general population with access to treatment and, on the other hand, the right to self-determination. The ethical principles of beneficence and non-maleficence [44] seem to be opposed. A way for managers and staff to handle this problem seemed to be to accept the situation and adapt to the older persons’ changed behavior and needs. However, the risk is that differential diagnoses such as depression, a brain tumor or hearing, and vision impairments are missed because of a diagnostic overshadowing [8]. If people with an ID are not diagnosed, there is a risk that the staff will not receive support and guidance from the healthcare, i.e., memory clinics, which they need. Staff in the disability service need support, guidance, and education because very few have health education and/or previous experience in the care of people with ID and dementia.

It would be desirable to get people with an ID to express their thoughts and wishes by themselves, which would illuminate the process from the first appearance of signs of dementia. However, since this implies methodological difficulties, staff and managers in this study can, in light of their several years of experience in working with older people with IDs, be considered their representatives. Descriptions in the interviews of staff and managers who together have extensive experience may possibly give a somewhat idealized picture of the situation given their knowledge about dementia and other common health problems in aged people with an ID. This needs to be taken into consideration when reading the results. However, extensive narrations as a basis for the analysis in this study and the overall experience of the interviewees counts as validity. Dementia among people with an ID was not the main question in the interview guides, but it became the clear concern that needed to be raised. If dementia had been the main focus of the interview, more aspects of dementia could have appeared. However, there were important experiences in the data provided in this study, which will hopefully contribute to the field of knowledge about people with an ID and dementia, but further research about the whole care pathway is needed.

The context of group homes and daily activity centers is organized in similar ways and, based upon a strong entitlement law known as the LSS act, it is based on the core values of autonomy, influence, and participation [22]. The culture in these two organizations, therefore, do not differ to any larger extent in Sweden, which means that the result is perceived to have transferability to the Swedish context and also to the Norwegian context due to a similar law. However, the transferability to other countries need to be considered carefully from the following perspectives: the organization of intellectual disability services, laws, and disability policy as well as the competence of managers and staff in each country. The similarities between most of the world’s countries is the implementation of human rights that protect the rights of vulnerable groups in societies [23].

## 5. Conclusions

This study presents results expressed as a model based on three themes about resources for the identification process of signs of suspected dementia. The importance of a caregiver relationship based on time and continuity is the basic resource for collegial and manager support as well as the consultation of specialists outside the intellectual disability service. Only when these resources are used will it be possible for staff to identify, and react to, dementia symptoms in a safe and knowledge-based way, initiate examination, design well-adapted care, and, if necessary, consider moving the person to adapted housing. A close collaboration with specialists within dementia care may contribute to a good working environment for staff with skilled guidance and support, continuous education, and the initiation of intervention research concerning the best accommodation form and digital support for people with an ID and staff.

The results are important for policy making in their ambition to improve the care for this vulnerable group. More focus needs to put on teaching staff about dementia among older people with ID since this important area is insufficient in medical and nursing education today. Further qualitative research may include those with ID and family members’ experiences of early signs as well as the staff’s experiences, which means that they acquire deeper knowledge of detecting and distinguishing signs of dementia over time and exploring the type of living standards and the environment, i.e., stay in place or specialized environment. Knowledge from these studies needs to be translated into interventions of integrated care for people with ID and dementia based on the complexity that this type of intervention implies.

## Figures and Tables

**Table 1:** Actors and patterns of core content in the three levels in the model (Figure 1) of resources for the identification process of signs among people with an ID and suspected dementia.

Level: Themes	Actors and Patterns of Core Content
First level: A close caring relationship provides reflection and understanding	Actors in this part of the caring process consist of the person with an ID and staff.
	Time and continuity in the care relationship are crucial to identify and respond to signs of a change in cognitive ability and thus indicate dementia.
	Staff must strive to understand the person’s changed expression and observed behaviors.
	Internal reflection, seeking to understand what the changes lead to, and how treatment and care can best be adapted.
Second level: A team relationship provides a basis for knowledge, support and continued action	Actors in this second part of the caring process consist of staff and managers in their own workplace.
	Necessary to obtain support from colleagues.
	Transfer experiences and reflections from the long-standing and ongoing process together with the person with an ID to colleagues and managers in recurring meetings.
	Staff express how managers have a responsibility and an opportunity to provide support based on their knowledge and experience.
Third level: External relations support examination and more adapted care	Actors on this third level consist of, beside the staff and managers in the ID-service, external staff from other municipal units, primary health care, and specialist care.
	Managers pave the way for contact with external resources and initiate an investigation for suspected dementia.
	Staff who know the older person with an ID accompany them to the office of the physician.
	In cases when the staff needs more knowledge about dementia and specialist support, dementia nurses are consulted.
